# The complete mitochondrial DNA sequence of Kashgarian loach (*Triplophysa yarkandensis*) from Bosten Lake

**DOI:** 10.1080/23802359.2020.1715881

**Published:** 2020-01-24

**Authors:** Xia Ning, Yan-Zhen Zhang, Zhi-Hai Sui, Xian-Qing Quan, Hai-Guang Zhang, Ling-Xiao Liu, Qing-Dian Han, Yun-Guo Liu

**Affiliations:** a Jinan Zhangqiu District Center for Disease Control and Prevention, Jinan, China;; b College of Life Sciences, Linyi University, Linyi, China;; c Linyi Academy of Agricultural Sciences, Linyi, China

**Keywords:** Kashgarian loach, *Triplophysa yarkandensis*, mitochondrial genome

## Abstract

*Triplophysa yarkandensis* is a specific cobitidae species that is endemic to Xinjiang Tarim River basin, China. The complete mitochondrial genome sequence of *T. yarkandensis* from Bosten Lake was determined in this study (Accession number MN821008). The mitogenome (16,552 bp) consists of 22 tRNA genes, 2 ribosomal RNA genes, 13 protein-coding genes, and 1 control region (D-loop region). The complete mitochondrial genome sequence of the *T. yarkandensis* provides an important data set for further study in genetic mechanism and classification.

As a kind of small economic fish, which belongs to *Triplophysa*, Nemacheilidae, Cypriniformes, Actinopterygii, Osteichthyes, *Triplophysa yarkandensis*, also called Kashgarian loach, is a specific omnivorous Cobitidae species that is only distributed in Xinjiang Tarim River basin, China (Chen et al. 2016). Most of them are just finger thick with bearded and scaleless, which is a relatively large individual species in the genus of *Triplophysa*. Due to the gradual depletion of water resources in Tarim River and the backward cultivation technology, *T. yarkandensis* is facing the danger of extinction (Gong et al. 2016). Here, the complete mitochondrial genome of *T. yarkandensis* was sequenced and characterized in detail. Kashgarian loach sample was collected from Bosten Lake (41°73′N, 86°95′E), a relatively closed water area, Xinjiang, China in July 2019. The specimen of Kashgarian loach, named as TriyarBost-01 was stored in the College of Life Sciences, Linyi University, Linyi, China. Total genomic DNA was extracted from *T. yarkandensis* muscle according to Liu et al. (2012, 2016). Then, the complete mitochondrial genome was sequenced using a shotgun approach and assembly. Subsequently, DNA sequence was analyzed using MEGA 7 (Kumar et al. 2016) and protein-coding genes were analyzed by ORF Finder (http://www.ncbi.nlm.nih.gov/gorf/gorf.html) using the invertebrate mitochondrial code. The tRNA genes were identified using ARWEN (Laslett and Canback 2008) and tRNA-scan SE (Lowe and Eddy 1997).

The complete *T. yarkandensis* mitochondrial genome (Accession number MH MN821008) was 16,552 nucleotides long, of which 15,557 nucleotides are coding DNA and 965 nucleotides are non-coding DNA. The mitochondrial genome of *T. yarkandensis* was found to contain 22 transfer RNA genes, 2 ribosomal RNA genes, 13 protein-coding genes, and 1 control region. The arrangement and composition of the mitochondrial genome are also comparable to other vertebrates (Liu and You 2015; Xu et al. 2015; Shan and Liu 2016; Li, Liu, Sui et al. 2019; Li, Liu, Zhang 2019). A phylogenetic tree was constructed based on the comparison of the complete mitochondrial genome sequences with other Cobitidae species using the neighbour-joining method (Figure 1). There were 13 intergenic spacers (total 73 bp) and 7 overlapping regions (total 27 bp) among the genes. The overall base composition was 27.21% A, 28.21% T, 19.03% C, and 25.55% G, with an AT content of 55.42%. To investigate the nucleotide bias, skew for a given strand was calculated as (A–T)/(A + T) or (G–C)/(G + C) (Perna and Kocher 1995). The AT and GC skews for the *T. yarkandensis* mitochondrial genome were −0.018 and 0.146, respectively; this finding indicated that the strand that encoded genes contained more T and G than A and C and this skew was evidence of codon usage bias. All 13 protein-coding genes identified in the *T. yarkandensis* mitochondrial genome started with the common initiation codon ATG, except for *COX1* and *ND6*, which began with GTG and TTA, respectively. For termination codons, *ND1*, *ND5,* and *COX1* terminate with TAG, whereas *ATP8*, *ATP 6,* and *ND4L* terminate with TAA, *ND6* terminate with CAT, respectively. In addition, the six remaining genes terminate with incomplete stop codon T-, that T- is the 5′ terminal of the adjacent gene, which presumptively formed a complete stop codon by post-transcriptional polyadenylation (Anderson et al. 1981). All the mitogenome genes were encoded on the H strand except for *ND6* and eight tRNA genes (*tRNA-Gln, Ala, Asn, Cys, Tyr, Ser, Glu, Pro*). The 12S rRNA (951 bp) gene and 16S rRNA (1678 bp) gene were located between the *tRNA-Phe* and *tRNA-Leu* genes and separated by the *tRNA-Val* gene. The lengths of 22 tRNA coding genes range in size from 69 to 75 bp. In addition, the control region was located between *tRNA-Pro* and *tRNA-Phe* with a length of 892 bp. Thus, the data would facilitate further investigations of phylogenetic relationships within Nemacheilidae.

**Figure 1.**
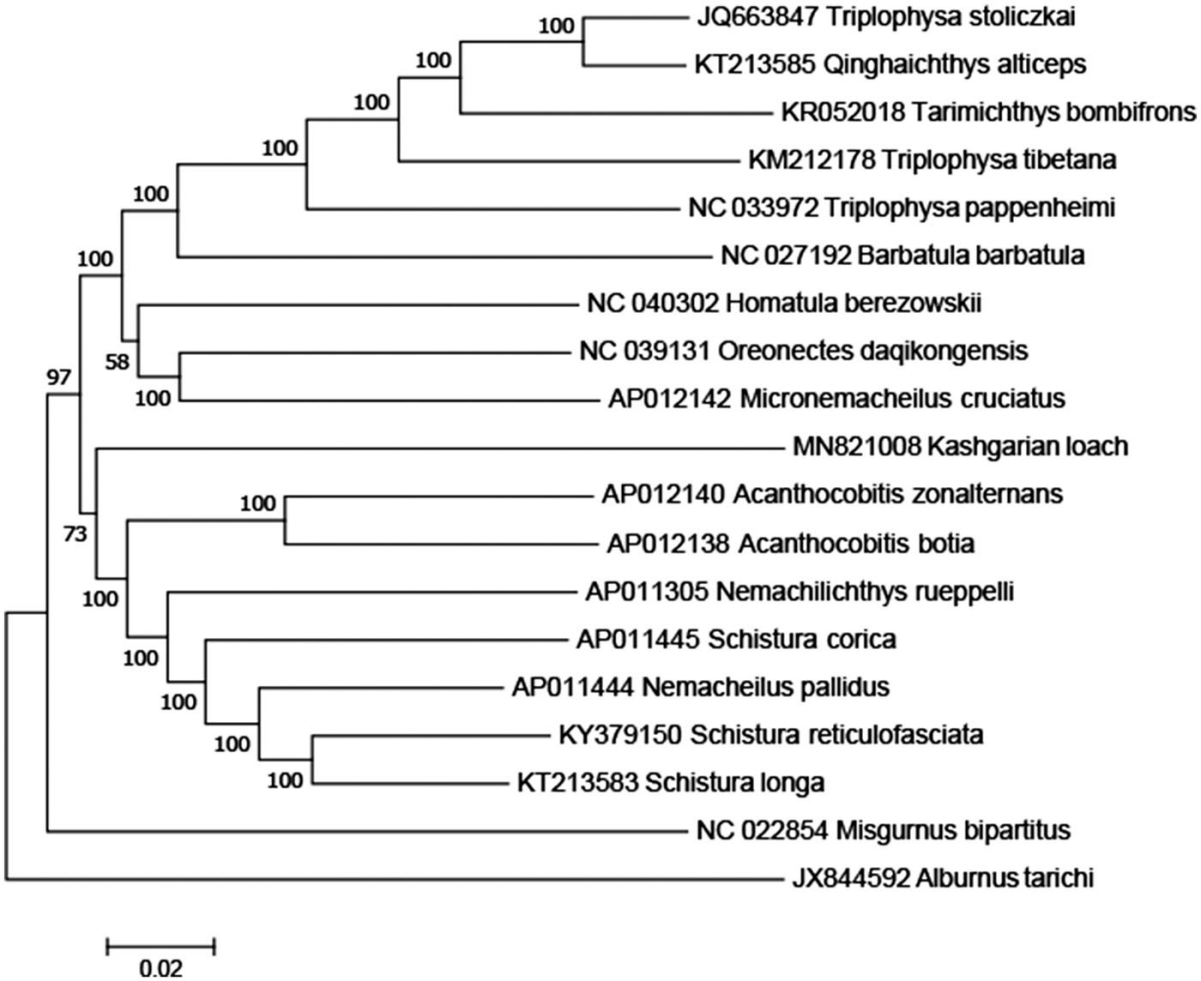
A phylogenetic tree constructed based on the comparison of complete mitochondrial genome sequences of the Nemacheilidae, *T. yarkandensis* (Kashgarian loach) and other 16 species of Nemacheilidae family. They are *Triplophysa stoliczkai*, *Qinghaichthys alticeps,* and *Triplophysa tibetana* (Tibetan stone loach), *Tarimichthys bombifrons*, *Barbatula barbatula*, *Oreonectes daqikongensis* and *Nemachilichthys rueppelli* (stone loach), *Triplophysa pappenheimi* (Yellow River stone loach), *Homatula berezowskii*, *Micronemacheilus cruciatus*, *Acanthocobitis zonalternans*, *Schistura longa* and *Schistura corica* (ray-finned loach), *Acanthocobitis botia* (Thailand mottled loach), *Nemacheilus pallidus* (undertone loach), *Schistura reticulofasciata* (stream loach). *Misgumus bipartitus* and *Albumus tarichi* are used as outgroup. Genbank accession numbers for all sequences are listed in the figure. The numbers at the nodes are bootstrap percent probability values based on 1000 replications.
